# Perceptions of factors influencing the introduction and adoption of electronic immunization registries in Tanzania and Zambia: a mixed methods study

**DOI:** 10.1186/s43058-020-00022-8

**Published:** 2020-03-30

**Authors:** Samantha B. Dolan, Mary E. Alao, Francis Dien Mwansa, Dafrossa C. Lymo, Ngwegwe Bulula, Emily Carnahan, Emily Beylerian, Laurie Werner, Jessica C. Shearer

**Affiliations:** 1 Dolan Consulting LLC, PATH, Seattle, USA; 2 Department of Global Health, University of Washington, Seattle, USA; 3 Johns Hopkins University, Bloomberg School of Public Health, Baltimore, USA; 4 Department of Public Health, Ministry of Health, Lusaka, Zambia; 5 Immunisation and Vaccines Development, Ministry of Health, Community Development, Gender, Elderly and Children, Dar es Salaam, Tanzania; 6 PATH, Seattle, USA

**Keywords:** Health systems, mHealth/eHealth, Electronic immunization registry, Scale-up, Mixed methods, Immunization, Digital health intervention, Adoption, Sustained use

## Abstract

**Background:**

As technology has become cheaper and more accessible, health programs are adopting digital health interventions (DHI) to improve the provision of and demand for health services. These interventions are complex and require strong coordination and support across different health system levels and government departments, and they need significant capacities in technology and information to be properly implemented. Electronic immunization registries (EIRs) are types of DHI used to capture, store, access, and share individual-level, longitudinal health information in digitized records. The BID Initiative worked in partnership with the governments of Tanzania and Zambia to introduce an EIR at the sub-national level in both countries within 5 years as part of a multi-component complex intervention package focusing on data use capacity-building.

**Methods:**

We aimed to gather and describe learnings from the BID experience by conducting a framework-based mixed methods study to describe perceptions of factors that influenced scale-up of the EIR. Data were collected through key informant interviews, a desk review, EIRs, and health management information systems. We described how implementation of the EIRs fulfilled domains described in our conceptual framework and used cases to illustrate the relationships and relative influence of domains for scale-up and adoption of the EIR.

**Results:**

We found that there was no single factor that seemed to influence the introduction or sustained adoption of the EIR as many of the factors were interrelated. For EIR introduction, strong strategic engagement among partners was important, while EIR adoption was influenced by adequate staffing at facilities, training, use of data for supervision, internet and electricity connectivity, and community sensitization.

**Conclusions:**

Organizations deploying DHIs in the future should consider how best to adapt their intervention to the existing ecosystem, including human resources and organizational capacity, as well as the changing technological landscape during planning and implementation.

Contributions to the literature

- Electronic immunization registries (EIRs) are complex interventions that require strong coordination and support across stakeholders to be successfully introduced and adopted by end users.
- Our comprehensive assessment found that many of the factors perceived to have influenced EIR scale-up are interrelated and largely reflect the existing health system challenges and enabling ecosystem, despite large upfront investments made by partnerships between global health non-government organization and governments.
- Considerations should be made for how best to adapt these types of interventions to existing ecosystems in terms of resource needs, capacity building, organizational factors, and changing technological landscapes.

## Introduction

Digital health interventions (DHIs) have become more prolific across health programs in low- and middle-income countries (LMICs) in recent years [1–4]. As technology has become cheaper and more accessible, health programs are adopting DHI to improve the provision of and demand for health services. These interventions have substantial potential to alter how health information is managed and utilized to improve health care and treatment. The World Health Assembly recognized the importance of digital technologies to support health systems in 2018 and urged member states to assess and consider how DHIs could be optimized and integrated into existing health systems [5].

However, despite the increased availability of technology, in LMICs there hasn’t been a corresponding increase in DHIs taken to regional or national scale. Projects may suffer from “pilotitis” and simply have been deployed to demonstrate feasibility or were not built to sustain increasing client volumes and do not have the resources to scale and become institutionalized [6, 7]. These interventions are complex; they require strong coordination and support across different health system levels and government departments and need significant capacities in technology and information to be properly implemented [8, 9]. Factors leading to acceptance and use of these interventions have been documented at small scales, but because there are few large-scale implementations at the facility level across geographic areas, more evidence is needed on factors influencing scale-up of digital solutions in LMICs [10].

Electronic immunization registries (EIRs) are types of DHI used to capture, store, access, and share individual-level, longitudinal health information in digitized records [11]. The use of DHIs by immunization programs has been encouraging for the uptake and adherence to vaccination schedules. For example, improvements in vaccination coverage were observed following the introduction of an EIR in Vietnam [12, 13]. The BID Initiative (http://bidinitiative.org/), led by PATH and funded by the Bill and Melinda Gates Foundation, worked in partnership with the governments of Tanzania and Zambia to introduce an EIR at the sub-national level in both countries within 5 years as part of a multi-component complex intervention package that focuses on data use capacity-building.

We were interested in understanding how the introduction of an EIR can be implemented effectively by the national governments, and how this strategy could be adapted for all immunizing health facilities beyond the pilot phase. The BID Initiative provided a unique opportunity to assess what factors influence the scale-up, or introduction and adoption, of DHIs in two settings as implemented through non-government organization and government partnerships. Scale-up refers to “deliberate efforts to increase the impact of innovations successfully tested in pilot or experimental projects so as to benefit more people and to foster policy and program development on a lasting basis” [14]. Introduction refers to a facility being sensitized and trained on the intervention package, while adoption refers to those facilities consistently using or maintaining the intervention package. For the purposes of this study, we consider only the introduction and adoption of an EIR in each facility within a region during the rollout of the intervention package, as precursors to creating sustainable systems. We aimed to describe the perceived facilitators and barriers as well as trends in introduction and adoption of EIRs in each country from the BID experience by conducting a mixed methods study with an embedded case study, within the Initiative’s evaluation.

## Intervention description

The BID Initiative partnered with the Ministries of Health (MOH) in Tanzania and Zambia to address key challenges in immunization data collection, quality, and use beginning in 2013. The areas of concern identified were as follows:

- incomplete or untimely data;
- inaccurate or uncertain denominators for calculating immunization rates;
- difficulty identifying infants who do not start immunization or who drop out (defaulter tracing);
- lack of unique identifiers;
- poor data visibility into supplies at the facility-level to district-level;
- complex data collection forms and tools;
- insufficient supply chains and logistics management; and
- inadequate data management and use capacity at all levels of the health system.

The BID Initiative implemented an intervention package to address the challenges identified, including establishment of user-advisory groups to provide input, development of the tablet-based EIR software with online and offline functionality that enables automated simplified reports (for more details see reference [15]), development of supply chain tools, provision of targeted supportive supervision for health workers, establishment of peer support networks (via WhatsApp groups), and creation of a data-use culture. By 2018, the package was introduced in the regions of Arusha, Dodoma, Kilimanjaro, and Tanga of Tanzania, and the Southern Province of Zambia. Project staff used a phased rollout approach to introduce the intervention package to each district. Completing paper-based forms and reports remained a requirement by the MOH throughout the project; therefore, all facilities completed dual data entry from the time of EIR introduction and onwards.

This study focuses on the introduction and adoption of the EIRs, recognizing however that other components of the intervention package contributed to system adoption. The EIR allows healthcare workers (HCWs) to register children, record vaccinations administered, quickly identify vaccinations due, and generate aggregate facility-level reports that feed into the health management information system (HMIS). In both countries, the intention of the EIR was to replicate and eventually replace the use of paper-based data collection tools.

## Methods: conceptual framework, data collection, and analysis

### Study design

We designed a framework-based mixed methods study aiming to describe perceptions of factors that influenced scale-up of the EIR in Tanzania and Zambia. Qualitative data were collected through key informant interviews and a desk review. Quantitative data from the EIRs and HMISs were used to describe facility characteristics and inform our qualitative findings by drawing conclusions about factors influencing EIR introduction and adoption. We aimed to describe how implementation of the EIRs fulfilled domains described in our conceptual framework and used district cases to illustrate the relationships and relative influence of domains on introduction and adoption.

### Conceptual framework

**Fig. 1:**
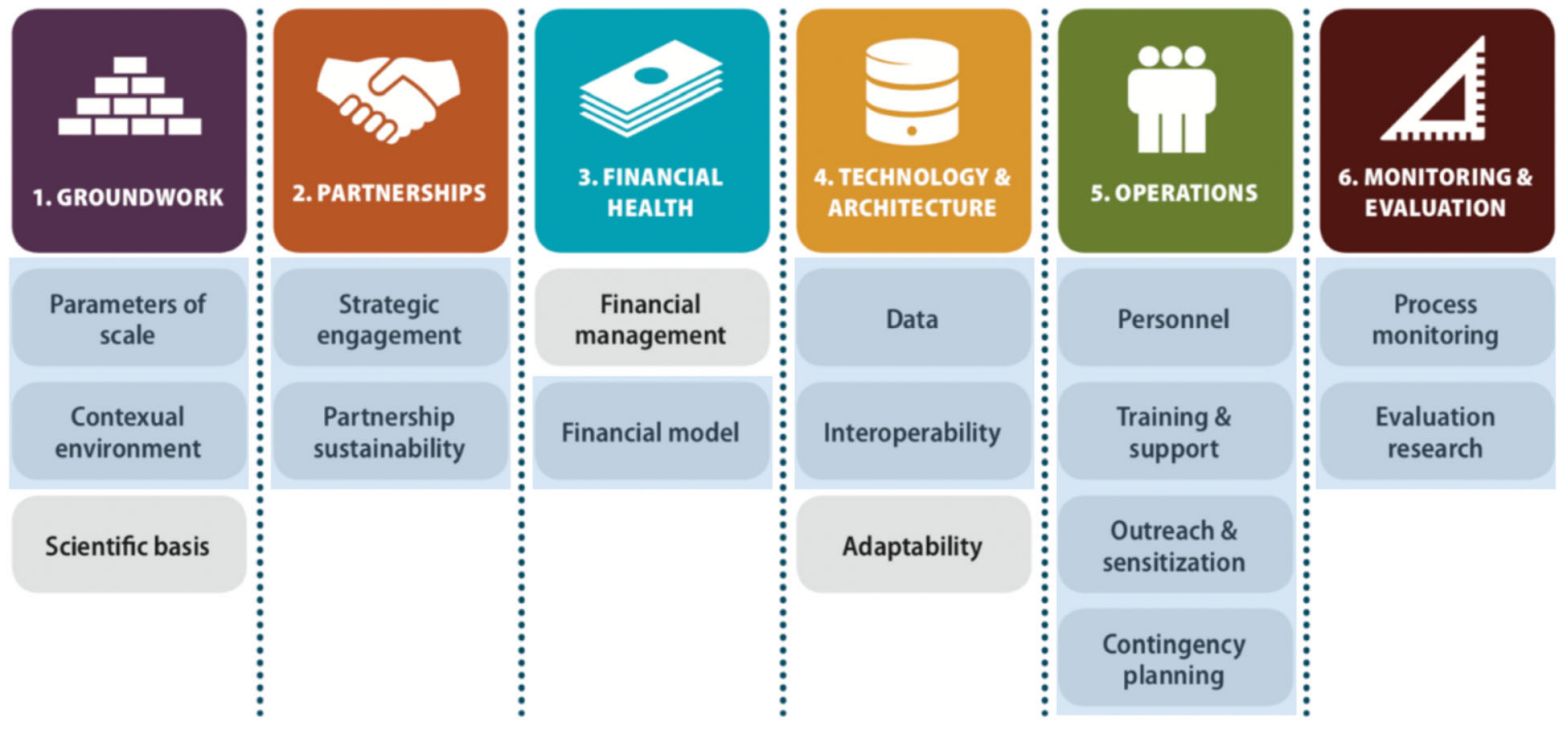
Axes and domains of the mHealth Assessment and Planning for Scale (MAPS) Toolkit (reproduced) [14]. Domains shaded in blue were included in the assessment. For definitions of each of the axes and domains, please refer to the MAPS Toolkit

We used the axes and domains outlined by the mHealth Assessment and Planning for Scale (MAPS) Toolkit as the conceptual framework to guide the study’s data collection approach, interview questions, and analytic framework as depicted in Fig. 1 [14]. The Toolkit was developed as a semi-quantitative approach for project managers to prospectively assess their programs’ readiness for scale. We applied the Toolkit retrospectively to describe the existence of MAPS’ domains during the scale-up processes and how fulfillment of the axes and domains differed across countries and low- and high-performing districts. Axes include groundwork, partnerships, financial health, technology and architecture, operations, and monitoring and evaluation (M&E). To narrow the project’s scope, we excluded domains that referred to the selection and design of the EIR software to focus on system level factors. Additionally, the financial health domain was modified as we recognized that these projects were heavily dependent on donor funding, and so assessing financial management would not necessarily provide actionable results for other countries. The framework was agreed upon by PATH staff involved with project implementation.

### Data collection

We performed a desk review to identify all project documentation to assess whether MAPS’ domains were considered during planning and implementation. Documentation was manually coded by MAPS’ domain by MA and reviewed by SD to classify whether each domain area was included in the document, the depth of inclusion was not captured, and data were collected, summarized, and categorized by country. Domains not covered in the reviewed documents were prioritized in the key informant interview questions. Results of the desk review were summarized by the number of MAPS’ domains covered by each type of document. We considered inclusion of each MAPS’ axis, and its subsequent domains, as indication of successfully planning or implementing scale-up.

We purposively sampled 14 key informants by role and level of involvement with the project among MOH staff at regional/provincial and district levels that could provide insightful and diverse information, along with PATH staff involved with the implementation of the intervention package. District staff were selected by EIR performance among facilities in their district, one staff each from a low- and high-performing district were chosen based on the capacity of district immunization officers to provide support and supervision, as perceived by PATH staff. The interview team (SD and MA) conducted semi-structured telephone interviews between September and October 2018 with key informants; one interviewee provided written responses. A translator was used for one interview during the call to help facilitate the discussion. Interview questions were adapted from the MAPS Toolkit (see Supplementary Material); questions differed by role of interviewee with separate sets of questions for MOH regional/provincial level staff, PATH staff, and MOH district level staff. The interview team took notes on each interview during the call, notes were manually coded by SD and MD, and code discrepancies were discussed until a consensus was reached. Themes were coded by MAPS’ domain. All interview notes were safeguarded on password protected computers by the interview team.

We summarized health facility characteristics on EIR use, staffing, location, and type of facility by province/region using data from HMIS, including the EIRs. We measured EIR introduction by the number of facilities with at least one record entered into the system. EIR adoption was measured by system use, calculated by the proportion of weeks, a facility entered data into the EIR, from the time the EIR went live to the last data pull (June 2018); data from the last month were excluded due to data quality concerns. The median and interquartile ranges of EIR use were calculated by district for each region using Tableau (Seattle, WA, USA). Counts of the number of facilities, mean values, and corresponding standard deviations were used to summarize facility characteristics within each province or region using Stata (StataCorp LLC, College Station, TX, USA). Facility types were categorized based on government definitions; dispensaries (in Tanzania) and health posts (in Zambia) were the lowest level of health service provision, health centers provide a wider range of services, and district or regional hospitals offer inpatient, outpatient, and specialized services [16].

As part of routine program evaluation, this study was determined as non-human subjects research by PATH. The interview team received permission from staff in the local governments to discuss project scale-up for program improvement purposes in the two countries. SD, MA, and JS led the design, implementation, and interpretation of findings for this study and were not involved in the BID Initiative design or implementation.

## Results

For the desk review, all documents related to BID from PATH’s files were reviewed, including evaluation reports, presentations, communications materials, briefs, and webpages. Of the available documents, 23 were deemed to be related to introduction and adoption of the intervention and were reviewed and categorized. All MAPS’ domains were discussed in at least one document, with the project resource documents covering all domains (see Table 1). These documents focused on designing the intervention package, evaluation results of the Initiative’s intervention activities, along with communication materials that described the on-going work, lessons learned from each country’s experience implementing the package, an external evaluation report, and presentations on project updates. No single document covered all domains.

**Table 1:** Coverage of MAPS Axes and Domains by Document Type and Country, proportion of sub-domains covered

		Tanzania		Zambia		Tanzania and Zambia	
Axis	Domain	Internal reports	Communication materials	Internal reports	Communication materials	Internal reports	Project resources
Groundwork	Domain 1. Parameters of scale	4/4	3/4	4/4	3/4	3/4	4/4
	Domain 2. Contextual environment	3/3	1/3	2/3	1/3	2/3	3/3
Partnerships	Domain 4. Strategic engagement	3/3	3/3	3/3	3/3	3/3	3/3
	Domain 5. Partnership sustainability	5/5	2/5	2/5	2/5	5/5	5/5
Financial health	Domain 7. Financial model	0/3	0/3	0/3	0/3	0/3	3/3
Technology and architecture	Domain 8. Data	1/4	1/4	1/4	1/4	1/4	4/4
	Domain 9. Interoperability	2/2	0/2	2/2	0/2	2/2	2/2
Operations	Domain 11. Personnel	1/2	0/2	0/2	0/2	1/2	2/2
	Domain 12. Training and support	3/4	1/4	3/4	2/4	0/4	4/4
	Domain 13. Outreach and Sensitization	0/2	0/2	0/2	0/2	1/2	2/2
	Domain 14. Contingency planning	0/2	0/2	0/2	0/2	0/2	2/2
Monitoring and evaluation	Domain 15. Process monitoring	2/3	0/3	2/3	0/3	0/3	3/3
	Domain 16. Evaluation research	7/7	1/7	7/7	1/7	2/7	7/7

MAPS’ domains 3, 6, and 10 were excluded from the study

### Trends in EIR use

**Fig. 2:**
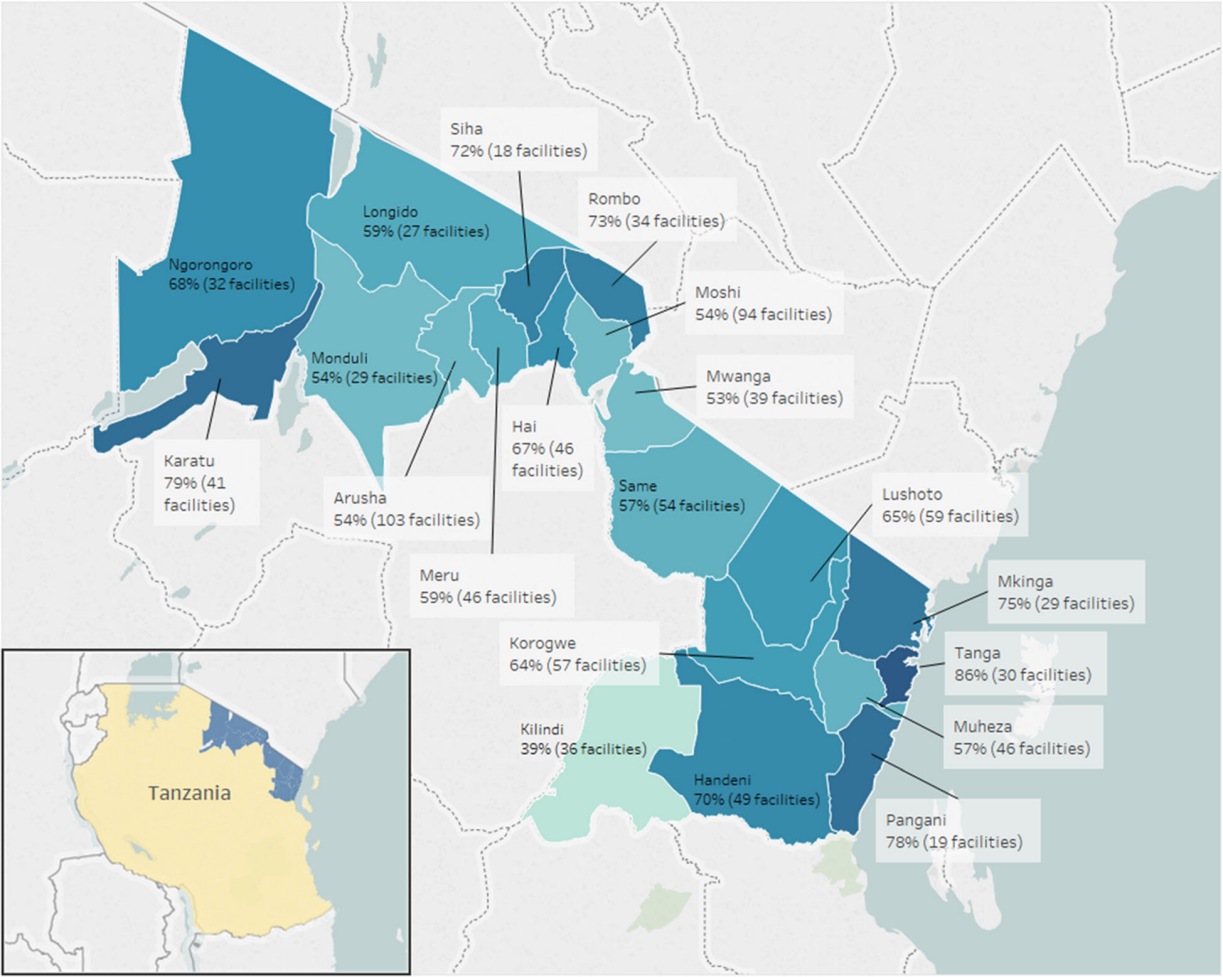
Average percentage of weeks active in EIR per facility by district, Tanzania, 2016-2018

**Fig. 3:**
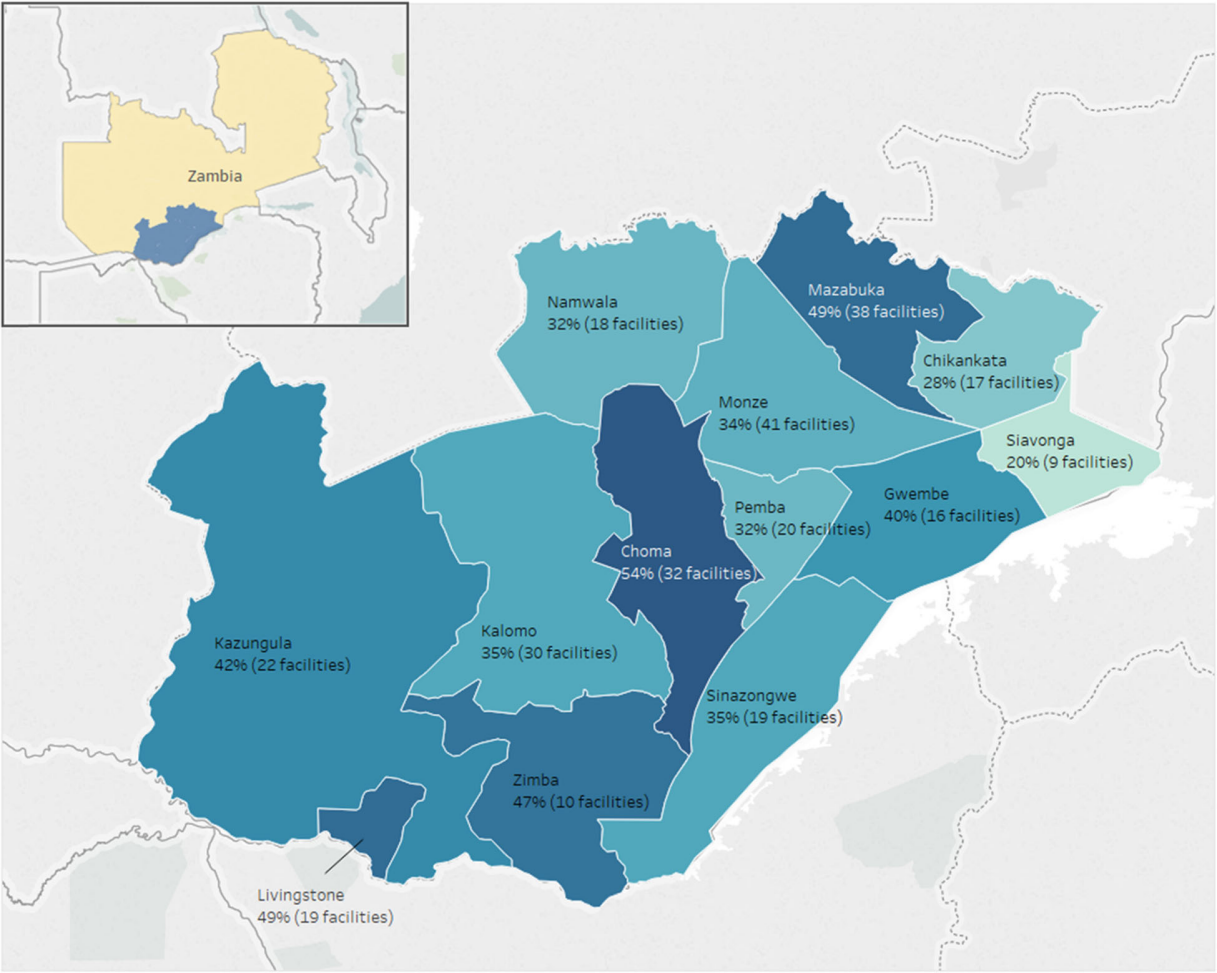
Average percentage of weeks active in EIR per facility by district, Zambia, 2017-2018

We analyzed data from 905 active facilities in Tanzania and 302 facilities in Zambia (see Table 2). Among facilities in Tanzania, the median EIR use over active-weeks was 71% for Arusha, 64% for Kilimanjaro, and 73% for Tanga, ranging from 39–86% per districts (see Fig. 2). In Zambia, the median percent of active-weeks using the EIR by facility was 40%, ranging from 22–59% per district (see Fig. 3).

**Table 2:** Characteristics of health facilities using the electronic immunization registry (EIR) in Tanzania (TZ) and Zambia (ZA), by region and province

Characteristics	Arusha, TZ	Kilimanjaro, TZ	Tanga, TZ	All Regions, TZ	Southern Province, ZA
Number of districts	6	6	8	20	13
Number of facilities	283	292	330	905	302
Number of patients	137,130	35,084	89,740	261,954	96,617
Time since EIR Introduction^5^	27 months	9 months	14 months	NA	14 months
EIR use percentage weeks active post-rollout, median [IQR]	71% [32%, 86%]	64% [40%, 80%]	73% [42%, 87%]	70% [39%, 87%]	40% [24%, 59%]
Primary power source, n (col%) ^1^					
Grid	102 (37%)	226 (79%)	-	328 (36%)	123 (41%)
Solar	87 (31%)	22 (8%)	-	109 (12%)	5 (2%)
None	11 (4%)	-	-	11 (1%)	144 (48%)
Number of HCWs per facility, mean (SD)^2^	2 (1)	2 (1)	3 (1)	2 (1)	-
Distance to regional DHO, km, mean (SD)	40 (32)	63 (174)	23 (14)	41 (102)	49.4 (27.5)
Ownership type, *n* (col %)^3^					
Private—FBO	90 (32%)	70 (25%)	40 (12%)	199 (22%)	-
Public—Government	181 (65%)	201 (71%)	279 (86%)	661 (74%)	-
Facility type, n (col %)^4^					
Dispensary/health post	218 (78%)	226 (79%)	282 (87%)	726 (82%)	47 (16%)
Health center	47 (17%)	45 (16%)	36 (11%)	128 (14%)	219 (73%)
Hospital	13 (5%)	14 (5%)	8 (2%)	35 (4%)	6 (2%)

Amongst those facilities that have input at least one record into the EIR

^1^Facilities missing data on primary power source: in Arusha (*n* = 78), Kilimanjaro (*n* = 37), and Tanga (*n* = 327); in Southern Province (*n* = 30), percentages will not add up to 100%

^2^Missing data regarding average number of HCWs in facilities in Southern Province, Zambia, percentages will not add up to 100%

^3^Facilities missing data on ownership type: in Arusha (*n* = 7), Kilimanjaro (*n* = 14), and Tanga (*n* = 8); in Southern Province (*n* = 302), percentages will not add up to 100%

^4^Facilities missing data on facility type: in Arusha (*n* = 0), Kilimanjaro (*n* = 14), and Tanga (*n* = 36); in Southern Province (*n* = 31), percentages will not add up to 100%

^5^Number of months since introduction of the first EIR system in at least one facility within a region or province to the time the key informant interviews were conducted (September 2018); TZ Introduction Dates: Arusha—June 2016, Kilimanjaro—December 2017, and Tanga—July 2017; Zambia Introduction Dates: July 2017; it took from 1 to 12 months for the EIR to be rolled out to all facilities within each region

### Perceptions of factors leading to successful introduction and adoption

Perceptions of facilitators and barriers to EIR introduction and adoption based on the key informant interviews are summarized in Table 3. We interviewed 12 of the 14 selected key informants; two informants could not be interviewed due to incompatible schedules with the study timeline. For each country, informants included two MOH provincial level staff from either the immunization program or the M&E unit, two MOH district level staff, and two PATH staff. We present the results by axis of the MAPS Toolkit.

**Table 3:** Perceptions of Facilitators and Barriers to Scale-Up of an Electronic Immunization Registry

Axis	Domain	Facilitators	Barriers
Groundwork,	Domains 1–7	• Identification of a long-term strategy • Conducted landscape analysis • Pilot EIR in one region • Support by MOH for electronic data	• Lack of electricity in facilities
Partnerships, and			
Financial health			
Technology and contingency planning	Domains 8, 9, and 14	• Accessibility of data at facility and district levels • Secure access to EIR • Use of data standards • Interoperability of EIR with stock management system (TZ) • Policies for lost or stolen tablets	• Delays with data synchronisation across the system • Discrepancies in data across systems • Inability to access data at provincial level • Lack of interoperability of EIR with HMIS (ZA) • Multiple versions of software used (ZA) • Limited funding and delays with equipment procurement
Operations-training	Domain 12	• Completed multiple training visits per facility • Trained HCWs and district staff to act as mentors and to provide technical support • Use of training checklists • Identified champions • Trained multiple staff cadres	• Limited MOH staff capacity to conduct mentorship and training • Limited time available for training • Inability to scale training approach • Accommodating varying skill levels and staff turnover
Operations-supervision and technical support	Domain 12	• Support of partner organisations for conducting supervision • Integration of supervision with existing structure • Use of supervision checklist and plans • Use of data to target problematic areas • Trained district staff to provide technical support • Creation of help desk	• Reliance on partner organisation for support • Integrated supervision can limit time spent addressing EIR issues • Limited funding to do EIR-specific supervision visits • Need for data access and dashboards (ZA) • Limited internet access • Lack of a contingency plan (ZA)
Operations-personnel and outreach	Domains 11 and 13	• HCW and MOH buy-in to EIR use • Supportive leadership • Inclusion of MOH and local leaders with all decision-making • Capacity to deploy program • Community sensitization • Support of partner organisation	• Multiple electronic systems deployed at facilities • Lack HCW skill and confidence • Limited staffing • Lack of involvement of technocrats with planning • Weakened leadership because of reliance on partner organisation • Lack of focus on sustained use of the EIR
Monitoring and evaluation	Domains 15 and 16	• Tracking indicators of use • Roll-out approach • Planning and review meetings • WhatsApp groups • Monitoring visits	• Used monitoring visits of other programs to observe EIR (ZA) • Few resources for monitoring • Lack of system interoperability (ZA) • Lack of indicators to track system maturity
Sustainability-other	NA	• Improved ability to register and track children • Creation of data use culture • Identification of partners • Continuity of internet connectivity	• Lack of support for data bundles, tablets, and system maintenance • Limited mentorship and leadership • High level of government involvement needed • Need for planning for scale-up alongside other programs

*TZ* Tanzania, *ZA* Zambia; the axes of groundwork, partnerships, and financial health were grouped, and operations was disaggregated by domain due to the amount of information collected

#### Groundwork, partnerships, and financial health (axes 1–3)

##### Facilitators

Prior to the introduction of the EIR, groundwork activities included identification of a long-term strategy and landscape analyses of the policy, technical, and electronic health environments. The analyses provided a contextual assessment of the existing capacities and challenges of implementing an EIR, and informed intervention customization. In both countries, the MOHs demonstrated an interest in shifting to increased use of electronic data, and therefore supported EIR introduction.

##### Barriers

It was noted that lack of electricity in some health facilities was an initial barrier. In Tanzania, 36% of facilities received power from an electric grid, but 12% relied on solar power or had no power source (see Table 2). In Zambia, many facilities had no primary power source (48%), while 41% were connected to the electric grid. Lack of electricity was resolved through the provision of solar panel kits. Both countries piloted the EIR in one region/province to ensure that the intervention worked and was acceptable before rolling-out to other areas.

#### Technology and contingency planning (axes 4 and 5)

##### Facilitators

**Fig. 4:**
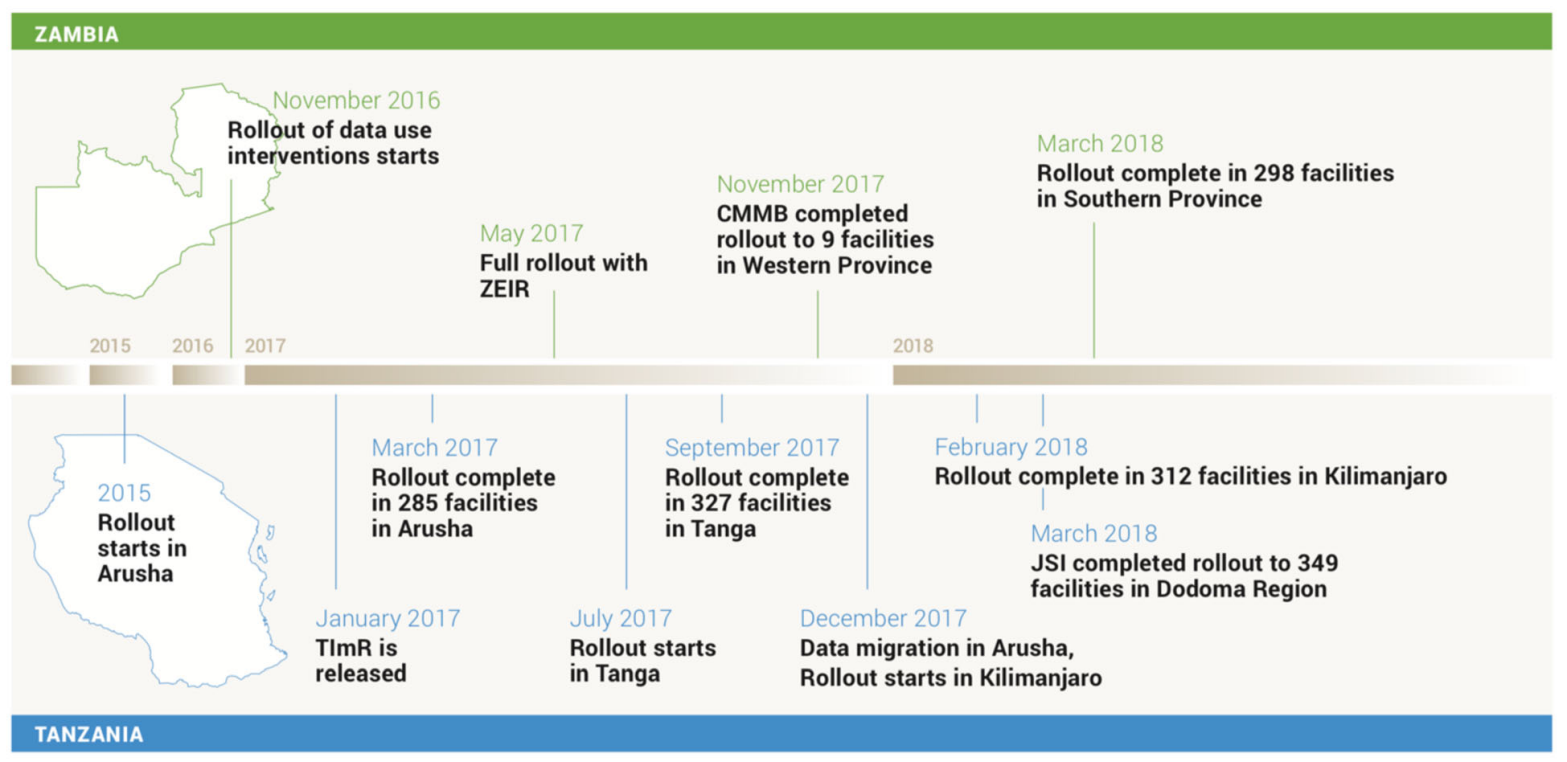
Timeline for EIR Introductions. CMMB Catholic Medical Mission Board, JSI John Snow, Inc., TImR Tanzania Immunization Registry, ZEIR Zambia Electronic Immunization Registry

In both Tanzania and Zambia, MOH and M&E staff expressed that EIR data accessibility was good at the district and facility levels. Data quality was perceived to have improved due in part to EIR data standards and interoperability with other systems. For example, M&E staff in Zambia reported that the EIR system rejected nonsensical data, and staff in Tanzania expressed that they were able to monitor errors and discrepancies between systems. In an effort to eliminate parallel systems, Tanzania’s EIR was integrated with a stock management system and Zambia’s EIR was being considered for integration with the HMIS. System security was ensured by only granting selected staff user credentials and passwords, and strong policies were in place for replacement of lost or stolen tablets. In Tanzania, government policy required that all client data be hosted locally (rather than in a cloud-based server), resulting in plans for utilization of Gavi (a private-public global health partnership) funding to procure more servers. In Tanzania, the first version of the EIR was initially deployed in Arusha in 2015 and was then replaced by an improved system, subsequently introduced to Tanga and Kilimanjaro in 2016–2017 (see Fig. 4). Rollout of the improved system throughout Arusha, Dodoma, Kilimanjaro, and Tanga was completed in February 2018. For Zambia, the rollout of the EIR began in one district in July 2017 and was completed in March 2018.

##### Barriers

Technological challenges occurred in Tanzania due to a 24–48-h delay in EIR data synchronization. Some users became discouraged by synchronization problems between the stock management system and EIR, possibly leading to drops in EIR use. Also, the staff that did not know how to check their data bundle usage were not syncing their EIR routinely, in consideration for saving data. Despite noted improvements in data quality, some staff in Tanzania saw discrepancies between their EIR and stock management system data, but these differences were expected because of synchronization issues and immaturity of the software. In Zambia, data were only accessible for review at the district level. As a result, MOH M&E staff could only review data on-site, which prohibited their ability to monitor sites in real time. Efforts were being made to create versions of the EIR dashboards specifically for provincial staff. In Zambia, software updates caused different system versions to be used in different facilities, which affected troubleshooting and record synchronization.

#### Operations—training (axis 5)

##### Facilitators

Two approaches were used to deploy the EIRS, training-of-trainers (TOTs) and on-the-job (OTJ) training. Initially, PATH staff conducted OTJ trainings consisting of three to four visits per facility over several months; the first visit introduced the project, the second focused on training and interaction with the system, and the third or fourth was used for strengthening HCW skills. Later, PATH staff led a TOT for district immunization officers who then led OTJ training for facility HCWs, while PATH staff provided coordination and support. Trained HCWs acted as mentors and champions to help train staff in other districts during scale-up. One district focal staff was trained to review data on a daily basis, help train staff, respond to the WhatsApp group, and ensure the EIR was seen as a government product. The training plan changed over time and varied by the level of individual need, as staff found it easier to train HCWs familiar with technology. This change management approach helped build local capacity and empowered MOH staff to use the system. District staff felt that they were well prepared for the introduction of the program, and that PATH staff were readily available to provide support. PATH staff in Zambia used a checklist with talking points to ensure training was consistent and complete. District staff appreciated that training was provided for nurses and other cadres, such as community health workers, who could help with continuity of EIR use during every immunization session and staff turnover. Both countries found the training strategy to be adequate and user-friendly.

##### Barriers

In both countries, more MOH staff were needed for system introduction because there were initially not enough staff with the capacity to conduct mentorship and training. PATH used a phased training approach in Tanzania to accommodate limited implementation staff. Training was initially conducted by PATH staff, followed by a TOT with district staff, which allowed for more expansive training. Later, this approach was replaced by TOT-mentor training that allowed mentors to more broadly assist with and enhance trainings. Some informants felt training was too short and that there was a need for ongoing HCW training and coaching to sustain EIR use. For busy facilities, training had to occur after hours or be rescheduled. Additionally, not all facilities and districts had motivated mentors and champions. District staff felt it was difficult to train HCWs because of their varied abilities and the need to continuously orient new staff due to high staff turnover rates.

#### Operations—supervision and technical support (axis 5)

##### Facilitators

In both countries, supervision visits were integrated within the existing MOH supervision structure to reduce new activities. Partner organizations, including PATH, helped support supervision visits and paid for mentor transport and per diem, which helped remove the burden from MOH. In Tanzania, PATH staff advocated for use of a supervision checklist with EIR indicators to track performance and help standardize the implementation process. At the district level, a comprehensive supervision plan was created that used mentors to provide monthly hands-on guidance. Supervision improved when MOH staff could view EIR reports to identify facility needs, emphasizing the importance of data accessibility for monitoring activities. As part of PATH’s approach to training and capacity building in Tanzania, 5–10 staff in each district were trained to provide technical support; a help desk was made available, in addition to a WhatsApp group, which included PATH, national and district staff, along with facility HCWs. When issues were too complex for district staff to tackle, issues were escalated to the partner software developers.

##### Barriers

Staff mentioned that using an integrated supervision approach had challenges, supervisors had to check on multiple systems, lacked EIR training, or had limited time to address key issues. District staff needed more supervision support to provide OTJ training and more funds for transport to the facilities to encourage system usage. Because of constrained government funding, PATH was still providing technical support in some districts beyond their planned timeline. In Zambia, data access and dashboards were needed by the district and provincial levels to monitor and identify struggling facilities. There were many facilities without connectivity in both countries, where server and internet access were problematic. In Zambia, it felt as if there was no contingency plan and that district teams did not have the capacity to sustain the project, which may have influenced buy-in and acceptance of the EIR. Additionally, there were some delays with equipment procurement and training due to competing priorities of the government.

#### Operations—personnel and outreach (domains 11 and 13)

##### Facilitators

Generally, HCWs appreciated the EIR and felt it reduced their workload and improved data reporting and quality, in the absence of parallel reporting systems. In both countries, national and regional/provincial level MOH leadership was supportive and crucial for decision-making about EIR deployment. Leaders understood the importance of the intervention and wanted to see it scaled-up. PATH staff ensured that the MOH, members of the national technical working group, local leaders, and all levels of EIR users were involved with decision-making. Stakeholders were engaged through the user advisory groups to ensure the EIR was appropriate for all user types. For example, in Tanzania, the user advisory group moved to abandon community registration activities because village leaders did not view it as their responsibility, and it was not cost-effective. In Zambia, the community health workers helped orient the community to the project, and local leaders were sensitized to increase EIR buy-in. To encourage community mobilization, posters were provided at the facilities, and HCWs were trained to address caregiver concerns. It was noted that scale-up went well because PATH was involved at every stage of the process.

##### Barriers

In terms of barriers to scale, other electronic systems were used in parallel with the EIR in some Zambian facilities, which sometimes overwhelmed HCWs. Most HCWs were not confident tablet users and some were slow learners or had technophobia; therefore, needing additional training. Among HCWs adequately using the EIR, some needed more capacity building around data use. Some HCWs felt the EIR added to their workload and was especially challenging for facilities with limited staff or high staff turnover. At the regional/provincial level in Tanzania, staff may have lost motivation because the system did not meet their demands, and they saw their workload increase, mainly due to the lack of EIR integration with HMIS. Staff from Tanzania felt MOH project leadership was challenging because of their reliance on PATH, for instance it was unclear who would train HCWs without PATH. Some informants felt project planning could have been improved if more technocrats were involved. PATH staff mentioned that is was challenged to work with MOH staff accustomed to short-term donor funding because they only focused on immediate deployment and not on sustained use of the system.

#### Monitoring and evaluation (domains 15 and 16)

##### Facilitators

MOH staff would review immunization coverage, defaulter rates, and stock levels as indicators of use of the system. To monitor program implementation, PATH staff met weekly to provide progress updates and plan for the way forward; MOH staff at all levels were made aware of updates, which helped with continuity of activities. To track facility progress, PATH staff in Zambia conducted monitoring visits using standardized tools and reporting mechanisms, while in Tanzania no monitoring data were available initially, so HCWs were asked about EIR usage; this was later replaced by weekly reports generated by the EIR. WhatsApp groups were also used to monitor EIR usage; facilities would report the number of children immunized. District staff used other program visits, like vaccine distribution, to check on EIR use. Facility data and updates were discussed at quarterly regional meetings and annual performance review meetings in Tanzania. In Zambia, the district management teams reviewed facility performance monthly, and at the lower levels, neighborhood health committees supervised community health workers and reported to the district.

##### Barriers

In Zambia, it was difficult for the provincial level to monitor the system because staff did not have access to the data (until recently), so they had to resort to in-person visits when monitoring other programs. Generally, it was felt that resources for monitoring were unavailable and therefore monitoring was weak. Because of limited provision of data bundles, district staff could not access all electronic systems and had to prioritize which system to use for routine monitoring. In Zambia, district staff were not able to compare EIR to HMIS data, due to lack of integration of the systems. PATH had to monitor the project due to too few resources and capacity available to the MOH. In Tanzania, PATH staff had planned for scale but did not track all indicators related to system maturity as they had seen done in other countries. No comprehensive analysis has been done to evaluate the system, and the countries are relying on PATH to do this.

#### Sustainability—other

##### Facilitators

The goal of national EIR scale-up in Tanzania and Zambia speaks to the need for ensuring system sustainability. Informants in both countries expressed approval of the BID Initiative in delivering user-friendly EIR systems that addressed many of their key challenges and areas of concern in immunization data. Staff felt that the EIR improved their immunization service performance, particularly in their ability to register and track children. In addition, the EIR fostered a data use culture in Zambia, as the HCWs expressed joy in being able to produce and view tables and graphs that allowed them to identify gaps in the continuum of immunization services. Identification of supporting partners was mentioned as a factor to facilitate scale-up. For example, staff in Tanzania shared that they were working with John Snow, Inc. (JSI) at district and regional levels and PATH supported scale-up in the Dodoma region, while the Catholic Medical Mission Board is leading the implementation of the EIR in the Western Province of Zambia. Partnerships will be valuable for scaling-up beyond the current province and regions.

##### Barriers

MOH staff expressed that there is a need for partners to support the technical environment through acquisition of data bundles in all facilities, procuring tablets, and maintaining the system. In Zambia, informants mentioned how partners lobbied telecommunication companies to make short message service (SMS) free for another project; they felt something similar should be done to get free data/air-time for their EIR. Informants expressed that they would tell their counterparts in other countries, whom may be interested in implementing and scaling EIRs, that it requires a high level of government involvement and prioritization beyond donor departure, strong leadership and decision-making, and planning for the scale-up of systems for other programs in addition to immunization.

**Table:** 

**Embedded case study- low- and high-performing districts** Two district staff were interviewed from each country, one from a low- and one from a high-performing district. Low- and high-performance was determined by the capacity of district immunization officers for providing support and supervision as observed by PATH staff. We summarized responses by district type*.* *Low-performing districts* Generally, low-performing districts found the EIR to be well received by HCWs and did not have different facilitators and barriers to scale-up compared with high-performing districts, but rather they faced challenges more severely when performing common activities. In both countries, it was felt that the project had **strong strategic engagement** among partners, as it was well supported by PATH and MOH leadership. We interviewed staff from one district where on average 40% of facilities used the EIR weekly, among 16 facilities, of which eight (50%) facilities were health centers and two (13%) were hospitals; five (31%) of the facilities were connected to the electric grid. **Lack of internet and electricity** were noted as common problems and impacted EIR users and **process monitoring** activities of district staff. Since most facilities did not have internet connectivity, HCWs had to travel to find internet to sync the EIR and obtain up-to-date **data**. It was mentioned that for outreach sessions and for large facilities multiple tablets were needed to prevent **data transmission** delays. Fortunately, staffing at the district and facility levels was not a problem, and the integrated approach for **supervision** worked well for the district; they believed this was due to the majority of staff being oriented to the EIR so that when they visited facilities, most staff were **trained** to check on EIR use. **Community sensitization** was seen as an important component that allowed for the EIR to be accepted and appreciated by caregivers and community leaders. The other district had on average 54% of facilities using the EIR weekly, among 103 facilities, of which 79 (81%) were dispensaries and 7 (6%) were hospitals; 40 (39%) facilities were connected to the electric grid. In the beginning, the EIR did not work well and the district relied on PATH to provide **technical support**. One of the biggest challenges facing the district was the shortage of **personnel** at facilities. There were few HCWs working in facilities during the EIR introduction of the EIR, which caused challenges with the uptake and continued use of the EIR. **Champions** were only used at the facility level, when they could also have also been used at the district level. District staff did not feel **training or supervision** were adequate, they felt there was not enough time to provide quality training outside of HCWs’ routine activities or to support HCWs during in-person visits. A virtual remote viewer was used to provide quick support to facilities and which allowed for remote rather than in-person **monitoring**; however, the viewer required both the district and the facility to have internet connectivity, which could not always be guaranteed. Staff used the EIR’s web application to review facility reports on the number of children that had been registered; however, the **lack of internet connectivity** became problematic, and staff were not always able to **access reports** in real-time. Because **more than one electronic system** was used at the district level, staff sometimes had to prioritize which of the systems to use with their **limited resources**. Additionally, district staff felt that facilities performing well, in terms of their use of the EIR, was because the HCWs **understood the value** of the system, while those that performed poorly was due to **understaffing**. *High-performing districts* Generally, staff from high-performing districts seemed to have more capacity for **providing mentorship, support, and supervision**. Similar to the low-performing districts, the district staff from high performing districts did not feel they had enough **resources** for **monitoring and trouble-shooting**, notably data bundles, airtime, and transportation. However, both districts did feel they had **adequate staffing levels** at the district and health facilities. In both countries, **mentors or champions** would visit facilities or **provide technical support** when district staff could not. Notably, the high-performing districts had a larger proportion of facilities **connected to the electric grid** than the low-performing districts. One of the high-performing districts had on average 54% of facilities using the EIR weekly, among 32 facilities of which 23 (72%) were health centers, and two (6%) were hospitals; 14 (44%) of facilities were connected to the electric grid. District staff felt it was helpful to have PATH staff **monitor** the EIR along with the **champions**. Multiple cadres of HCWs were **trained** to use the EIR to help build in-facility capacity. The district staff were afraid of placing the responsibility of tablet **security** on the facilities as it was not practical to have facilities replace tablets because of their expense. The other district had on average 67% of facilities using the EIR weekly, among 46 facilities, of which 38 (85%) were dispensaries and two (4%) were hospitals, 34 (70%) of facilities were **connected to the electric grid**. District staff mentioned that they leveraged the use of an assistant district officer to help **train mentors**, additionally **champions** were identified from existing immunization program staff and helped with **training** at other facilities. The champions were brought along during training and were motivational for other HCWs being trained. Mentors were included in **supervision visits** to oversee EIR issues; every month facilities were visited for hands-on guidance and to observe EIR use. It was mandatory for facilities to **report** how many children had been registered and immunized to the WhatsApp group. Additionally, the district signed a memorandum of understanding with an internet service provider to ensure that **data bundles** would be provided to facilities for the next year. District staff had received good feedback from the **community** that they felt services at the facility were being provided faster and records were well kept following introduction of the EIR.	

## Discussion

We illuminated some of the major perceptions of the facilitators and barriers influencing the scale-up of EIRs and observed these were similar for both countries. There was no single factor that seemed to influence the introduction or sustained adoption of the EIR as many of the factors were interrelated. For EIR introduction, strong strategic engagement among partners was important, while EIR adoption was influenced by adequate staffing at facilities, HCW training, use of data for supervision, internet and electricity connectivity, and community sensitization. The major barriers largely reflected the available human and financial resources, the level of training and capacity building needed, and existing health system challenges. Lack of internet and electricity were problematic and likely impacted adoption of the EIRs, as well as lack of personnel. It is likely that the burden of dual data entry impacted the adoption of the EIRs; however, this was not mentioned by key informants. The Tanzania MOH is looking to remove the requirement of paper-based reporting as they continue to scale-up the EIR to other regions. Problems with data synchronization delays are being addressed through further software integration enhancements and acquisition of more servers for the government data center. Our case studies mainly reflected the domains of the contextual environment, data, personnel, training and support, and process monitoring, with a close interplay between the availability of staff and accessibility of data for adequate monitoring and supervision. We found that the use of PATH for deployment and scale was both a facilitator and barrier, indicating the need for strong ownership over implementation while also planning for hand-off of activities to the MOH from the onset of a project. A list of recommendations for scale-up based on findings from this study can be found in Table 4.

**Table 4:** Recommendations on system sustainability

Axis	Domain	Recommendations
Groundwork,	Domains 1-7	• Understand the existing context, including the technical capacity, for implementing an EIR prior to introduction of the system • Strong government interest in electronic data is key to continued program support • Piloting and software updates should be included in planning for scale; a phased roll-out approach can help accommodate changes
Partnerships, and		
Financial Health		
Technology and contingency planning	Domains 8, 9, and 14	• EIR data should be easily accessed and used to monitor errors occurring at the facilities • Create mechanisms to ensure EIR security • Plan for the number of servers needed to host data locally
Operations-training	Domain 12	• Training strategies should accommodate a variety of skill levels, be standardized across facilities, include multiple cadres of staff, and incorporate multiple on-site visits • Ensure there are enough staff to perform high-quality training and leverage the use of mentors and champions at the facility-level • Create training plans to accommodate staff turnover
Operations- supervision and technical support	Domain 12	• Integrate supervision within existing structures and adequately budget for visits and incorporate capacity building • Use standardized supervision checklists • Develop technical support team to respond assist with troubleshooting and triage issues
Operations- personnel and outreach	Domains 11 and 13	• Incorporate all levels of EIR-users into program decision-making • Use community mobilization/sensitization to increase buy-in for the system • Consistently engage with partners at every step of the roll-out • Understand existing user capacity and incorporate this into training strategies • Plan for sustained use of the system from the beginning, including handover of activities from partner organizations to the government
Monitoring and evaluation	Domains 15 and 16	• Provide access to EIR data so supervisors can monitor the system remotely • Use standard indicators, tools, and reporting mechanisms to monitor the system • Develop plan for scale-up and indicators to track system maturity
Sustainability—other	NA	• Create a data use culture among users and supervisors • Identify partners that can support scale-up in other regions • Plan for equipment and data bundle costs as well as system maintenance, monitoring, and supervision activity costs • Encourage a high-level of government involvement and leadership • Plan for scale-up alongside other health programs

A unique aspect of this study was the use of the MAPS Toolkit, which allowed for comprehensive evaluation of the major components needed to introduce and adopt the EIRs as part of the scale-up plan. The EIRs deployed in Tanzania and Zambia were planned for scale prior to piloting the systems; this is a rare occurrence in LMICs where limited numbers of DHIs are scaled following a pilot or “proof-of-concept” deployment [6, 7, 17]. The variability in the success of EIR adoption is seen by the range of use amongst districts across each country. Despite the inclusion of all MAPS’ domains in existing program documentation, weaknesses emerged during scale-up of the EIRs suggesting that there may be factors that still prevent successful EIR adoption. We observed through our use of the Toolkit that there is perhaps a hierarchy of axes and domains or perhaps a third dimension of factors that should be considered for projects planning to scale. For instance, although introduction of the EIR to the targeted facilities could be considered successful, we observed how sustained use of the system varied, suggesting that the importance of each domain may differ by stage of system implementation and by level of the health system. Additionally, implementers should consider if all domains are sufficient or necessary at each stage of system maturity or implementation.

The EIR implementation challenges are not unique to immunization programs, our results closely reflect what has been found for other DHI. The enabling ecosystem, or external factors, can greatly influence the success of these types of interventions [6, 18]. An unpublished study has used data from the EIRs in Tanzania and Zambia to assess determinants of use and found significant differences by type of facility, district, and facility power source. For mobile health (mHealth) interventions in Malawi and Zambia, the major implementation challenges for SMS technology were mainly related to the program’s human resource and transportation needs, rather than being specific to the technology itself; the intervention brought more complexity to the existing system [19]. This was similar to what we found in terms of the ability of the MOH to support supervision and monitoring of the EIRs. Adequate stewardship of DHI programs has become better recognized as a pillar of successful interventions and if it is not institutionalized, can lead to inefficiencies and lack of country ownership [20]. The disadvantages of a DHI can become more evident when implemented in a low-resource setting as the choice of the intervention may be limited and falter from technological deficits, in addition to the need for creation and support of new job roles, responsibilities, and organizational partnerships [21, 22]. Therefore, despite some organizations advocating for EIRs as a solution to overcome data accessibility and quality issues, we found that these types of interventions are heavily dependent on the existing health system environment.

For the BID Initiative, the EIRs were designed and deployed with government involvement and used guidance and standards created as a collaborative effort amongst stakeholders; this was highly documented in our desk review, but was not mentioned during interviews. This strategy aligns with other studies showing that intervention scale-up is influenced by data and evidence driven support, along with how well the intervention aligns with the priorities of users and government needs [21, 23]. A systematic review of mHealth interventions in Africa found that successful interventions are due to adequately adapting the product to the local setting, having strong relevant stakeholder collaboration in addition to government involvement [8]. Additionally, mHealth interventions in South Africa were found to be successful when there was a supportive policy environment and well-developed information and communications technology industry [9]. As we also observed for the adoption of EIRs in Tanzania and Zambia, challenges to scale-up include competing interests, financing, maintaining growth, packaging the intervention to be context-specific, and engaging HCWs and communities in the process of moving from pilot to scale [21, 24].

Assessing scale may be premature if an intervention’s effectiveness and impact have not been evaluated. There is a need to understand the implementation context of a DHI and how this impacts project fidelity [25]. Findings from this study help to provide needed context to understand the degree of sustained use, which in turn influences the resulting immunization coverage. Successfully scaling up of a DHI is contingent on the intervention demonstrating an impact on health outcomes, which continues to remain a challenge as there are few studies that assess impact on clinical outcomes in LMICs [6, 17, 24]. Researchers and policy makers have recognized that an implementation science agenda is needed to help expand the breadth of relevant evidence available for DHIs, this includes conducting more mixed methods and quasi-experimental studies that can help understand the influence of contextual factors on implementation of an intervention [26]. These studies are necessary, but may not be sufficient to demonstrate impact on health outcomes. Using rigorous assessments alongside a strategic plan for scale-up that involves a high level of government commitment can ensure that future DHIs are sustained and impactful.

Our study was limited by MOH staff availability, as we were unable to obtain responses from heads of the immunization programs which we believed limited our ability to assess the MAPS’ domains related to governance, partnership sustainability, and the financial model of the project. The EIRs were deployed at different times in each country which could have influenced the relationship between use and scale, where we would expect to see those areas with longer use having fewer barriers to scale. In Tanzania, data from the Dodoma region were not available. Additionally, we did not assess the financial health of the BID project in each country as it was beyond the scope of this study. The cost of program ownership and operational costs is an important factor for scale-up, and as others have noted, has been a consistent gap in DHI evaluations and research [6].

## Conclusion

We described what it takes to deploy a DHI with a large upfront investment in development and deployment in two LMICs using a comprehensive evaluation framework and found that the perceptions of facilitators and barriers reflect the existing enabling environment, including the relevancy of policies, and the technological and workforce capacity. Key enablers of EIR adoption included adequate staffing, supervision, internet and electricity connectivity, and community sensitization, along with strong strategic engagement. Barriers of EIR adoption included lack of personnel and inadequate training and internet and electricity connectivity. Organizations deploying DHIs in the future should consider how best to adapt their intervention to the existing ecosystem, including human resources and organizational capacity, as well as the changing technological landscape during planning and implementation. To have an impact on health outcomes, EIR introduction and adoption have to be sustained at scale. As DHIs continue to evolve, they will need to better adapt to the existing resources, capacity, and changing technology landscape of countries looking to upgrade their health information systems.

## Supplementary information

**Additional file 1.** Key Informant Interview Questions.

## Data Availability

The datasets used and/or analyzed during the current study are available from the corresponding author on reasonable request. The notes of the interviews underlying the qualitative results presented will not be made publicly available as the participants did not consent to have these notes shared in full. If requested, excerpts from the interviews that are relevant to the study can be shared (contact sdolan11@uw.edu). The data underlying the quantitative results will not be made available as they are owned by the governments of Tanzania and Zambia, researchers who meet the criteria for access to confidential data can contact Dr. Dafrossa Lymo of Tanzania (dafrossac@gmail.com) or Dr. Francis Mwansa of Zambia (fmdien@gmail.com) to request access to the data.

## Abbreviations

DHI: Digital health intervention
EIR: Electronic immunization registry
HCW: Healthcare worker
HMIS: Health management information system
JSI: John Snow, Inc.
LMIC: Low- and middle-income country
M&E: Monitoring and evaluation
MAPS: mHealth Assessment and Planning for Scale
mHealth: Mobile health
MOH: Ministry of health
OTJ: On-the-job
SMS: Short message service
TOT: Training-of-trainers
